# R-CHOP方案和R-DA-EPOCH方案一线治疗non-GCB型弥漫大B细胞淋巴瘤回顾性研究

**DOI:** 10.3760/cma.j.issn.0253-2727.2022.04.014

**Published:** 2022-04

**Authors:** 孜岩 何, 文娟 俞, 珊珊 索, 敬翰 王, 海涛 孟, 文渊 麦, 菊英 韦, 敏 杨, 莉萍 毛, 洁 金

**Affiliations:** 1 杭州市临安区第一人民医院，杭州 311300 The First People's Hospital of Lin'an District, Hangzhou 311300, China; 2 浙江大学医学院附属第一医院，杭州 310003 The First Affiliated Hospital of Zhejiang University, Hangzhou 310003, China

弥漫大B细胞淋巴瘤（DLBCL）是最常见的非霍奇金淋巴瘤亚型，具有高度异质性，基于免疫组化的Hans分型分为生发中心来源型（GCB）和非生发中心来源型（non-GCB）。Alizadeh等[1]研究显示，同样使用R-CHOP方案治疗，GCB型DLBCL具有更长的总生存（OS）期，non-GCB型相对GCB型治疗难度大、预后差。本研究我们回顾性分析88例一线方案采用R-CHOP或R-DA-EPOCH治疗的non-GCB型DLBCL患者资料，比较两种方案的疗效及预后。

## 病例与方法

1. 病例资料：收集浙江大学医学院附属第一医院经病理及免疫组化确诊的初发non-GCB型DLBCL患者临床资料，选取一线方案采用R-CHOP或R-DA-EPOCH方案治疗至少3个疗程的患者，依照Ann Arbor分期标准进行临床分期。将2013年1月1日至2015年6月30日一线采用R-CHOP方案治疗的48例患者临床资料归为R-CHOP组，中位年龄59（20～78）岁，男24例、女24例，Ann Arbor Ⅰ～Ⅱ期19例、Ⅲ～Ⅳ期29例，国际预后指数（IPI）评分0～2分36例、3～5分12例。将2015年7月1日至2017年12月31日一线采用R-DA-EPOCH方案治疗的40例患者临床资料归为R-DA-EPOCH组，中位年龄48.5（24～71）岁，男21例、女19例，Ann Arbor Ⅰ～Ⅱ期9例、Ⅲ～Ⅳ期31例，IPI评分0～2分24例、3～5分16例。所有患者均行骨髓穿刺检查以明确有无骨髓侵犯。所有患者人类免疫缺陷病毒（HIV）阴性。

2. 治疗方法：R-CHOP方案：利妥昔单抗375 mg/m^2^，d 0；环磷酰胺750 mg/m^2^，d 1；表柔比星70 mg/m^2^，d 1；长春地辛2.8 mg/m^2^，d 1；泼尼松60 mg·m^−2^·d^−1^，d 1～5；每3周为1个疗程。R-DA-EPOCH方案：利妥昔单抗375 mg/m^2^，d 0；依托泊苷50 mg·m^−2^·d^−1^、长春地辛0.8 mg·m^−2^·d^−1^、表柔比星15 mg·m^−2^·d^−1^持续静脉滴注96 h；环磷酰胺750 mg/m^2^，d 5；泼尼松60 mg·m^−2^·d^−1^，d 1～5；每3周为1个疗程。IPI 4～5分患者常规行鞘内注射地塞米松及阿糖胞苷预防中枢神经系统侵犯。若规律化疗中无疾病进展，均化疗6～8个疗程。

3. 疗效评价：疗效判断主要依据全身PET/CT或CT检查结果，初诊时有骨髓侵犯的患者，评价疗效时包括治疗后骨髓形态学±病理学结果。疗效判断依据国际工作组 2014修订版标准分为完全缓解（CR）、部分缓解（PR）、疾病稳定（SD）和疾病进展（PD）。总反应率（ORR）为CR率+PR率。

4. 随访：治疗后半年内每月复查1次，之后1年半内每3个月复查1次，再后3年内每半年复查1次，5年后每年复查直至疾病进展。主要监测患者的血常规、LDH、淋巴系统彩超或CT及患者首诊时的不适症状有无再次出现等。随访截止日期为 2020年9月，中位随访55（33～92）个月。无进展生存（PFS）时间定义为开始化疗至有客观证据表明疾病进展的时间。总生存（OS）时间定义为开始化疗至死亡或末次随访时间。

5. 统计学处理：应用SPSS 23.0软件进行统计分析，计数资料组间比较采用卡方检验或Fisher精确概率法；采用Kaplan-Meier法绘制生存曲线，OS及PFS的组间比较采用Log-rank检验，*P*<0.05为差异有统计学意义。

## 结果

1. 近期疗效：88例病例均可评价疗效，其中CR 57例（64.8％）、PR 19例（21.6％）、SD 5例（5.7％）、PD 7例（7.9％），ORR为86.4％（76/88）。R-CHOP组和R-DA-EPOCH组CR率、ORR分别为70.8％（34/48）、89.6％（43/48）和57.5％（23/40）、82.5％（33/40），差异均无统计学意义（*P*值分别为0.192、0.367）。我们对其中BCL-2、BCL-6、CD5蛋白表达及骨髓是否受累资料完整的77例患者，以BCL-2≥50％、BCL-6≥30％、CD5>10％者为阳性进行分析，两种治疗方案在各临床因素中CR率差异均无统计学意义（表1）。

**表1:** R-CHOP方案和R-DA-EPOCH方案一线治疗77例DLBCL non-GCB型患者不同临床特征近期疗效比较

临床特征	R-CHOP组		R-DA-EPOCH组		*P*值
	例数	完全缓解［例（％）］	例数	完全缓解［例（％）］	
性别					
男	21	12（57.1）	17	9（52.9）	1.000
女	22	18（81.8）	17	11（64.7）	0.282
年龄					
>60岁	19	12（63.2）	7	2（28.6）	0.190
≤60岁	24	18（75.0）	27	18（66.7）	0.554
Ann Arbor分期					
Ⅰ~Ⅱ期	17	14（84.2）	8	8（100.0）	0.527
Ⅲ~Ⅳ期	26	16（61.5）	26	12（46.2）	0.404
IPI评分					
0~2分	31	23（74.2）	20	16（80.0）	0.743
3~5分	12	7（58.3）	14	4（28.6）	0.233
骨髓是否受累					
是	8	4（50.0）	6	3（50.0）	1.000
否	35	26（74.3）	28	17（60.7）	0.286
BCL-2表达					
阳性	33	24（72.7）	27	15（55.6）	0.186
阴性	10	6（60.0）	7	5（71.4）	1.000
BCL-6表达					
阳性	27	19（70.4）	28	17（60.7）	0.573
阴性	16	11（68.8）	6	3（50.0）	0.624
CD5表达					
阳性	10	7（70.0）	6	3（50.0）	0.607
阴性	33	23（69.7）	28	17（60.7）	0.590

注：R-CHOP：利妥昔单抗+环磷酰胺+表柔比星+长春地辛+泼尼松；R-DA-EPOCH：利妥昔单抗+依托泊苷+长春地辛+表柔比星+环磷酰胺+泼尼松；DLBCL：弥漫大B细胞淋巴瘤；non-GCB：非生发中心来源型；IPI：国际预后指数

2. 生存分析：R-CHOP组和R-DA-EPOCH组1年、3年OS率分别为（89.6±4.4）％、（83.3±5.4）％和（92.5±4.2）％、（89.9±4.8）％，1年、3年PFS率分别为（79.2±5.9）％、（68.8±6.7）％和（77.0±6.7）％、（63.5±7.8）％，组间比较差异均无统计学意义（*P*值分别为0.310、0.889）。单独分析58例年龄≤60岁患者生存情况，R-CHOP组和R-DA-EPOCH组1年、3年OS率分别为（92.3±5.2）％、（84.6±7.1）％和（90.6±5.2）％、（87.5±5.8）％，1年、3年PFS率分别为（80.8±7.7）％、（69.2±9.1）％和（74.8±7.7）％、（65.0±8.5）％，组间比较差异均无统计学意义（*P*值分别为0.794、0.826）。

## 讨论

DLBCL是一种生物异质性非常强的肿瘤，不同亚型的临床特征、治疗反应等方面表现不同，预后差异大。刘薇等[2]回顾性分析27例双表达淋巴瘤患者，其结外侵犯的比例为70.4％，non-GCB型占80.0％，预后更差。目前R-CHOP方案仍然是初治DLBCL患者的标准治疗方案。但R-CHOP治疗DLBCL IPI/年龄调整IPI（aaIPI）中高危患者疗效仍不理想，大于30％的DBLCL患者最终复发，大部分在治疗后早期复发，一部分患者在治疗后5年甚至更长时间复发，晚期复发患者即使初治时临床特征较好，但复发后仍然预后较差[3]。对年轻、aaIPI>1分的DLBCL患者，中国DLBCL诊断治疗指南[4]则推荐采用利妥昔单抗联合强化疗，EPOCH方案是提高化疗效果的一项新策略。早在2006年Julio等[5]一项前瞻性研究，对31例aaIPI评分在2～3分的DLBCL患者一线使用R-DA-EPOCH治疗，CR率为83.8％，PR率为12.9％，2年的无事件生存（EFS）及OS率分别为68％和75％。2013年R-DA-EPOCH在治疗原发纵隔（胸腺）大B细胞淋巴瘤（PMBCL）已得到普及。何靖等[6]报道DA-EPOCH（±R）方案治疗19例高危DLBCL患者，CR率为57.9％、ORR为84.2％。国外Pejša等[7]的一项回顾性研究对75 例预后不良的DLBCL患者予以R-DA-EPOCH方案化疗，CR、PR、PD 率分别为55％、25％、7％，患者的3年、5 年OS率分别为75％、70％，PFS率均为61％。本研究R-DA-EPOCH方案一线治疗non-GCB型DLBCL的CR率为57.5％（23/40）、ORR为82.5％（33/40），3年OS率为（89.9±4.8）％，PFS率为（63.5±7.8）％，与上述报道类似。杨萍等[8]回顾性分析2000年1月至2015年4月在北京大学第三医院就诊的122例年轻高危的DLBCL患者的临床资料，发现利妥昔单抗的应用及高剂量的化疗能够改善患者生存。但Wilson等[9]以年龄≥18岁、ECOG 0～2分的初治DLBCL患者为研究对象，比较R-DA-EPOCH（241例）和R-CHOP（250例）方案（21 d为1个周期共6个周期）一线治疗疗效与安全性，结果显示2年PFS率分别为78.9％、75.5％（*P*＝0.652），2年OS率分别为86.5％、85.7％（*P*＝0.641），3～5级不良事件发生率分别为16.9％、10.7％（*P*＝0.001），且根据年龄或IPI分类的各临床亚组，均不能从R-DA-EPOCH方案治疗中获益。Shah等[10]回顾性分析了年龄≥18岁的PMBCL患者分别接受R-CHOP（56例）与剂量调整后的R-EPOCH方案（76例）一线治疗，虽然剂量调整后的R-EPOCH方案提高了CR率，但毒副作用增加，其2年OS率分别为89％和91％，差异无统计学意义。上述文献显示，在非霍奇金淋巴瘤治疗中，与R-CHOP相比，R-DA-EPOCH方案化疗毒性更大，在改善患者的PFS或OS上并未显示明显优势。本研究一线治疗初发的non-GCB型DLBCL，R-CHOP方案和R-DA-EPOCH方案1年、3年OS及PFS率差异也无统计意义。即便年轻患者R-CHOP方案和R-DA-EPOCH方案1年、3年OS及PFS率差异也无统计意义。

除了Hans分型中的non-GCB，决定DLBCL不良预后因素还有很多。傅志英等[11]对525例DLBCL预后影响因素进行分析，认为性别、年龄、初期分期、B症状、LDH水平、是否使用利妥昔单抗治疗与DLBCL的预后存在关系。俞文娟等[12]回顾性分析223例DLBCL患者的临床资料，认为Myc/Bcl-2双表达及TP53高表达是DLBCL患者的独立预后不良因素，进一步分析发现TP53蛋白表达组non-GCB型居多。许亚茹等[13]对148例原发性DLBCL进行生存分析，认为年龄>60岁是预测DLBCL总生存和无进展生存的一个重要独立不良预后因素。方姝等[14]认为异常升高的β_2_微球蛋白是影响DLBCL患者OS的独立因素，aaIPI评分2～3分与初治骨髓受累的患者OS与PFS更差。有报道CD5阳性的DLBCL呈侵袭性病程，预后差[15]。我们的研究提示：两种治疗方案在不同的性别，年龄分组，Ann Arbor分期，IPI评分，骨髓是否受累及BCL-2、BCL-6、CD5蛋白表达高低的患者中CR率差异均无统计学意义，也可能与样本量小且所选病例均为non-GCB型有关。

因此，本研究提示，一线治疗初发non-GCB型DLBCL，R-DA-EPOCH方案可能并不优于R-CHOP方案，在年轻患者中亦未显示优势。
